# Repurposing the Natural Product Maslinic Acid for Combating *Clostridioides difficile* Infections

**DOI:** 10.1155/ijm/9364932

**Published:** 2026-11-02

**Authors:** Brice J. Stolz, Nader S. Abutaleb, Ahmed A. Abouelkhair, Mohamed N. Seleem

**Affiliations:** ^1^ Department of Biomedical Sciences and Pathobiology Virginia-Maryland College of Veterinary Medicine Virginia Polytechnic Institute and State University Blacksburg Virginia USA vt.edu; ^2^ Center for One Health Research Virginia Polytechnic Institute and State University Blacksburg Virginia USA vt.edu; ^3^ Department of Microbiology and Immunology Faculty of Pharmacy Zagazig University Zagazig Al-Sharqia Egypt zu.edu.eg

## Abstract

*Clostridioides difficile* infection remains a leading cause of healthcare‐associated diarrhea, and treatment options remain constrained by frequent recurrence and disruption of the intestinal microbiota. Natural products with selective antibacterial activity may provide new therapeutic opportunities for *C. difficile*. In this study, the pentacyclic triterpene maslinic acid, a naturally occurring compound abundant in olives and olive‐derived products, was evaluated for its antibacterial activity against *C. difficile*. Maslinic acid inhibited a diverse panel of clinical isolates, including hypervirulent strains, with MIC values of 8–16 *μ*g/mL while exhibiting substantially weaker activity against representative commensal gut bacteria. Time‐kill studies demonstrated complete elimination of detectable *C. difficile* within 2 h of treatment, indicating rapid bactericidal activity. Additionally, its antibacterial potency was preserved when tested at high bacterial inoculum and following prolonged exposure to simulated gastric and intestinal fluids, suggesting favorable stability under gastrointestinal conditions. Maslinic acid also showed no cytotoxicity to Caco‐2 cells at concentrations substantially exceeding its MICs. Mechanistic studies further indicated that maslinic acid preferentially interfered with protein and lipid biosynthetic pathways without evidence of membrane disruption. Moreover, evaluation of structurally related pentacyclic triterpenes identified several analogs with comparable activity with maslinic acid and highlighted structural features associated with antibacterial potency. Collectively, these findings identify maslinic acid as a selective, rapidly bactericidal natural product with characteristics that support its continued investigation as a potential therapeutic candidate for *C. difficile* infections.

## 1. Introduction

*Clostridioides difficile* is the leading cause of healthcare‐associated infectious diarrhea and remains a major source of morbidity and mortality worldwide [1]. In the United States alone, *C. difficile* accounts for approximately 400,000–500,000 infections and nearly 30,000 deaths annually, placing a substantial burden on the healthcare systems through prolonged hospitalization, increased medical costs, and frequent disease recurrence [2]. Although the incidence of *C. difficile* infections has declined modestly in recent years, hypervirulent strains continue to drive severe disease outbreaks and remain responsible for considerable healthcare‐associated transmission [3–5]. Moreover, healthcare costs related to *C. difficile* infections are exceedingly high, averaging approximately $5–6 billion per year in the United States [6]. Consequently, the Centers for Disease Control and Prevention (CDC) has labeled *C. difficile* as an urgent threat because of its severe impact on patients, rate of recurrence, and overall healthcare costs along with the potential for rapid and widespread transmission of disease [7]. These ongoing clinical challenges highlight the need for development of new therapeutics.

Initial treatment options remain limited, with vancomycin and fidaxomicin serving as the primary first‐line antimicrobial therapies. Both of which are characterized by low intestinal absorption and potent efficacy against vegetative *C. difficile* cells [8]. Metronidazole was previously widely used for treatment of *C. difficile* infections but is currently only utilized as a supplementary treatment or in extremely mild cases not at high risk for recurrent infection [9]. These recommendations are attributed to the fact that metronidazole is less efficacious compared with vancomycin where analyses of patient outcomes demonstrated inferior outcomes with moderate or severe *C. difficile* infections cases [10, 11]. Moreover, vancomycin and fidaxomicin outcomes have notably been marked with high rates of recurrence and treatment failure with rates as high as 30% and 20%, respectively [12]. There are other nonantibiotic therapies targeting *C. difficile* infections such as monoclonal antibodies directly inhibiting C*. difficile* toxins like bezlotoxumab and fecal microbiota transplants (FMT) [13]. Bezlotoxumab has no activity against acute *C. difficile* infections and has to be administered with either vancomycin or fidaxomicin [14]. Bezlotoxumab′s use is compromised primarily by its high cost and lack of efficacy in the clinic [15]. As of 2025, bezlotoxumab has ceased production, which further highlights changes in the therapeutic landscape for recurrent CDI [16]. FMT is currently an effective treatment option for recurrent *C. difficile* infections [17]. More recently, the FDA approved two microbiota‐based products (REBYOTA and VOWST), for the prevention of recurrent CDI following antibacterial treatment. Current American Gastroenterological Association guidelines support the use of FMT postantibiotic treatment of the second episode of recurrent CDI, although recommendations vary according to clinical circumstances and immune status [18]. FMT still cannot be administered to patients with prior complications such as severely immunocompromised patients and those having ulcerative colitis [18] and for patients under 18 years [19]. In light of the shortcomings of anti‐*C. difficile* infections therapeutics, discovery of novel, safe, and effective therapeutics remains critical.

In a previous screening to discover new anti‐*C. difficile* therapeutics, the natural product maslinic acid showed relatively potent activity [20]. Maslinic acid is fairly abundant in many edible plants and is commonly sourced as a byproduct of olive oil production. It was previously reported to have antiviral, antioxidant, and several other therapeutic effects [21–25]. Maslinic acid has favorable characteristics for *C. difficile* infections treatment as it possesses very low intestinal absorption, which is attractive for *C. difficile* infections treatment to accumulate at sufficient concentrations at the infection site, and is nontoxic in vitro and in vivo [26, 27]. This study will further evaluate maslinic acid′s potential use as a naturally available therapeutic for *C. difficile* infections. To build a more robust profile of its activity, killing kinetics, anticommensal activity against representative members of the normal intestinal microbiota, cytotoxicity and activity against high *C. difficile* inoculum, and after exposure to simulated gastrointestinal conditions were all tested. Additionally, the major biosynthetic pathways that are affected by maslinic acid treatment were explored via the macromolecular biosynthesis assay. Finally, the anti‐*C. difficile* activity of other natural pentacyclic triterpene compounds related to maslinic acid was evaluated.

## 2. Material and Methods

### 2.1. Bacterial Strains, Reagents, and Media

The bacterial strains used in this study (Table S1) were obtained from the Biodefense and Emerging Infections Research Resources Repository (BEI Resources; Manassas, Virginia), the American Type Culture Collection (ATCC; Manassas, Virginia), or the CDC (Atlanta, Georgia). All chemicals and reagents were purchased from commercial suppliers. Aanaerobic GasPak sachets (Becton, Dickinson and Company, Sparks, Maryland) were used for routine procedures and maintenance of anaerobic conditions during incubation. All experiments use Brain heart infusion broth (BHIS) (Becton, Dickinson and Company, Sparks, Maryland), supplemented with yeast extract (Fisher Scientific, Suwanee, Georgia), L‐cysteine (ThermoFisher Scientific, Waltham, Massachusetts), and vitamin K1, resazurin, and hemin (Sigma‐Aldrich, St. Louis, Missouri) for bacteria growth. Maslinic acid (Ambeed, Arlington Heights, Illinois), vancomycin (Gold Biotechnology, St. Louis, Missouri), fidaxomicin (Biosynth Carbosynth, San Diego, California), gatifloxacin and linezolid (Chem‐Impex, Wood Dale, Illinois), rifaximin (Sigma‐Aldrich, St. Louis, Missouri), triclosan and oxacillin (TCI America, Portland, Oregon), phosphate‐buffered saline (PBS; Corning, New York, New York) and the pentacyclic triterpene compound library are sourced from MedChemExpress Monmouth Junction, NJ). Simulated gastric fluid (SGF) and simulated intestinal fluid (SIF) were purchased from RICCA Chemical Company (Arlington, Texas, United States).

For mammalian cell‐based experiments, human colon adenocarcinoma cells (Caco‐2) were obtained from ATCC (Manassas, Virginia) and maintained using Eagle′s Minimum Essential Medium (EMEM; ATCC, Manassas, Virginia) supplemented with fetal bovine serum (FBS; Corning, Woodland, California).

### 2.2. Activity of Maslinic Acid Against a Panel of *C. difficile* Clinical Isolates

The minimum inhibitory concentration (MIC) of maslinic acid and comparator antibiotics against a panel of *C. difficile* clinical isolates was determined using the broth microdilution method as previously described [28]. Briefly, bacterial suspensions were adjusted to approximately 5 × 10^5^ CFU/mL and exposed to twofold serial dilutions of maslinic acid or comparator antibiotics in brain heart infusion supplemented broth (BHIS). Following anaerobic incubation at 37°C for 48 h, MICs were determined as the lowest concentrations preventing visible bacterial growth. MIC_50_ and MIC_90_ values represent the minimum concentrations required to inhibit 50% and 90% of the isolates tested, respectively. Experiments were performed in triplicate and repeated at least twice.

### 2.3. MICs of Maslinic Acid Against Representative Members of Commensal Gut Bacteria

Maslinic acid′s MICs against gut microbiota strains were evaluated as previously described [29, 30]. *Bacteroides* and *Bifidobacterium* strains were incubated on BHIS agar plates for 48 h anaerobically, whereas *Lactobacillus* strains were incubated on MRS agar at 37°C in presence of 5% CO_2_ for 48 h. Plates were incubated at either 48 h at 37°C in anaerobic conditions (*Bifidobacterium* and *Bacteroides*) or in the presence of 5% CO_2_ at 37°C (*Lactobacillus*) before visual determination of MICs.

### 2.4. Time‐Kill Kinetics Assay of Maslinic Acid

A time‐kill kinetics assay was performed as previously described [31, 32]. Logarithmic‐phase *C. difficile* ATCC 43255 cultures (~10^5^ CFU/mL) were exposed to maslinic acid, vancomycin, or fidaxomicin at the indicated concentrations. At predetermined intervals, aliquots were collected, serially diluted, and plated on BHIS agar to determine viable bacterial counts.

### 2.5. Cytotoxicity Assessment of Maslinic Acid Against Colonic Epithelial Cells

Caco‐2 colonic epithelial cells were utilized for a cytotoxicity assay to determine the potential toxicity of maslinic acid as previously performed [33, 34]. Cells were exposed to maslinic acid (32–128 *μ*g/mL) for 24 h, and viability was determined using the MTS assay. Cells treated with DMSO served as the vehicle control.

### 2.6. *C. difficile* Inoculum Effect on Maslinic Acid′s Activity

The efficacy of maslinic acid against high inoculum sizes of *C. difficile* ATCC‐BAA 1870 and 43255 was evaluated via the broth microdilution technique [35, 36]. A standard inoculum (5 × 10^5^) and higher inoculum sizes (~5 × 10^6^ and 5 × 10^7^ CFU/mL) of both *C. difficile* strains were prepared. Maslinic acid and respective controls were evaluated against each inoculum.

### 2.7. Effect of Gastric and Intestinal Fluids on Maslinic Acid′s Activity

To determine the effect of gastric and intestinal environments on the activity of maslinic acid, the MICs of maslinic acid and control antibiotics were determined after exposure to the SGF and SIF for 4, 24, and 48 h against *C. difficile* ATCC 43255 and ATCC‐BAA 1870.

### 2.8. Macromolecular Synthesis Inhibition Assay for Maslinic Acid

To assess the effects of maslinic acid on the essential cellular processes of *C. difficile* and to identify the potential molecular targets, the macromolecular synthesis inhibition assays were performed as described in a previous report [37]. Radiolabeled precursors were utilized to monitor the synthesis of key macromolecules as listed: [^3^H] thymidine (1 *μ*Ci/mL) for labeling DNA biosynthesis, [^3^H] uridine (0.5 *μ*Ci/mL) for labeling RNA biosynthesis, [^14^C] leucine (0.5 *μ*Ci/mL) for labeling protein biosynthesis, [^14^C] acetic acid sodium (0.5 *μ*Ci/mL) for labeling fatty acids biosynthesis, and [^14^C] N‐acetylglucosamine (0.3 *μ*Ci/mL) for labeling cell wall biosynthesis. A logarithmic culture of *C. difficile* ATCC 43255 (~1– 3 × 10^8^ CFU/mL) was washed with PBS and resuspended in prereduced BHIS broth. Then, the cultures were supplemented with the respective radiolabeled precursors before the addition of maslinic acid (0.5×, 1× or 2× MIC). Gatifloxacin, rifaximin, linezolid, triclosan, and oxacillin (2× MIC) were used as positive controls for inhibition of DNA, RNA, protein, fatty acid, and cell wall biosynthesis, respectively. Following anaerobic incubation at 37°C (for 4 h), samples from each tube were transferred into tubes containing ice‐cold 10% trichloroacetic acid (TCA) and kept on ice for 1 h to allow the radiolabeled material to precipitate. Afterward, the TCA‐precipitated radiolabeled macromolecules were filtered through GF‐75 glass microfiber filter (Sterlitech Corporation, Auburn, Washington) using a Millipore glass microanalysis filter holder and flask (Sigma‐Aldrich, St. Louis, Missouri) under vacuum. The precipitated macromolecules were washed twice with 10 mL of 5% TCA followed by 10 mL of 75% ethanol under vacuum to pull the wash liquid through. The glass filters were air‐dried and placed in scintillation vials before adding add 5 mL of Ultima Gold scintillation cocktail (Revvity, Waltham, Massachusetts). The TCA‐precipitated radioactivity was quantified by counting the filters in a liquid scintillation counter (Tricarb 5110TR, Perkin Elmer, Shelton, Connecticut) to obtain levels of radiolabeled material incorporated into aliquots. The data was presented as % of incorporated radioactivity standardized to the untreated group. The data were analyzed via one‐way ANOVA with post hoc Dunnet′s test for multiple comparison using GraphPad Prism software (GraphPad Software, San Diego, California). A *p* value of < 0.05 was considered statistically significant indicated with an asterisk ( ^∗^). Increasing levels of significance is indicated with more asterisks: *p* < 0.01 ( ^∗∗^) and *p* < 0.001 ( ^∗∗∗^).

## 3. Results

### 3.1. Anti‐*C. difficile* Activity of Maslinic Acid Against a Panel of Clinical Isolates

We initially discovered maslinic acid′s anti‐*C. difficile* activity by screening a compound library containing molecules with antiviral activity [20]. It showed somewhat weak activity against *C. difficile* (MIC = 16 * μ*M), but it is poorly absorbed from the gastrointestinal tract, which is desirable for *C. difficile* infections to achieve high concentrations at the site of infection. To further confirm the activity of maslinic acid against *C. difficile*, its activity was evaluated against 21 isolates of pathogenic *C. difficile.* Against the tested strains, maslinic acid displayed modest activity with MIC values ranging 8–16 *μ*g/mL. It inhibited 50% and 90% of the strains tested (MIC_50_ and MIC_90_), at the concentrations of 8 and 16 *μ*g/mL, respectively (Table 1). The control antibiotics vancomycin displayed an MIC range of 0.5–2 *μ*g/mL, with MIC_50_ and MIC_90_ values of 0.5 and 1 *μ*g/mL, respectively. The MIC_50_ and MIC_90_ values of fidaxomicin were 0.015–0.06 *μ*g/mL, 0.03 *μ*g/mL and 0.06 *μ*g/mL, respectively.

**Table 1:** MICs (*μ*g/mL) of maslinic acid versus a panel of *C. difficile* isolates.

*C. difficile* strains	MIC (*μ*g/mL)		
	Maslinic acid	Vancomycin	Fidaxomicin
ATCC 43255	8	0.5	0.015
ATCC‐BAA 1870	16	1	0.03
ATCC‐BAA 1871	16	2	0.06
ATCC 9689	8	1	0.015
ATCC 70057	8	0.5	0.015
ATCC 630	8	0.5	0.015
HM‐88	16	0.5	0.06
HM‐89	8	1	0.03
HM‐745	16	1	0.03
NR‐32888	8	1	0.03
NR‐32884	8	1	0.03
NR‐32897	16	0.5	0.06
NR 49288	16	0.5	0.03
NR 49308	8	0.5	0.015
NR 49310	16	0.5	0.015
AR 1078	8	0.5	0.015
AR 1081	16	1	0.03
AR 1082	16	0.5	0.03
AR 1087	16	1	0.015
AR 1093	16	0.5	0.03
AR 1094	8	0.5	0.03
MIC_50_	8	0.5	0.03
MIC_90_	16	1	0.06

### 3.2. Activity of Maslinic Acid Against Representative Commensal Gut Microbiota

Representative members of the human gut microbiota including *Lactobacillus*, *Bacteroides*, and *Bifidobacterium* species were tested against maslinic acid in an MIC assay (Table 2). Maslinic acid showed limited activity versus the representative members of the human gut microbiota with MIC values of 64 to > 128 *μ*g/mL. Contrarily, vancomycin and fidaxomicin significantly inhibited the microbiota strains tested. When tested against *Lactobacillus* strains, vancomycin did not show inhibitory activity (MICs > 128 *μ*g/mL), whereas fidaxomicin showed MIC values of 16–128 *μ*g/mL. Vancomycin also showed activity at higher concentrations (MICs = 16–32 * μ*g/mL) against *Bacteroides* strains and exhibited potent activity against *Bifidobacterium* strains inhibiting their growth at a concentration as low as 0.25 *μ*g/mL. Similarly, fidaxomicin inhibited *Bifidobacterium* strains at concentrations (MIC = 0.015 * μ*g/mL) similar to its MICs against *C. difficile.*

**Table 2:** MICs (*μ*g/mL) of maslinic acid and control antibiotics versus human gut microbiota.

Microbiota strain	Maslinic acid	Vancomycin	Fidaxomicin
*L. brevis* ATCC 14869	> 128	> 128	128
*L. rhamnosus* ATCC 53103	> 128	> 128	32
*L. casei* ATCC 334	> 128	> 128	16
*B. fragilis* HM‐709	128	32	> 128
*B. fragilis* HM‐714	128	32	> 128
*B. fragilis* HM‐710	64	16	> 128
*B. adolescentis* HM‐633	128	0.25	0.015
*B. breve* ATCC 15700	64	0.25	0.015
*B. breve* HM‐1120	64	0.25	0.015

### 3.3. Killing Kinetics of Maslinic Acid Against *C. difficile*

Maslinic acid and standard *C. difficile* antibiotic treatments were evaluated against *C. difficile* ATCC 43255 at 3× MIC. Maslinic acid demonstrated a rapid bactericidal activity, leaving no detectable colonies (below the limit of detection) after 2 h of incubation (Figure 1). Vancomycin and fidaxomicin reduced bacterial count by approximately 2 log_10_ CFU/mL within 6 h and showed complete clearance after 12 and 24 h, respectively.

**Figure 1:**
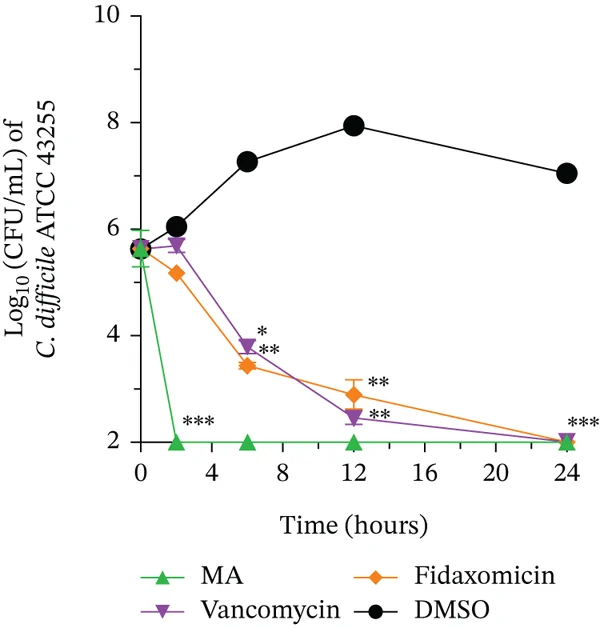
Time‐kill kinetics assay of maslinic acid versus *C. difficile* ATCC 43255. Bacteria were incubated with either maslinic acid (MA), vancomycin, fidaxomicin (at 5× MIC), or 2% DMSO (growth control). At each time point, an aliquot was taken and serially diluted onto BHIS agar plates. Data is presented as average log_10_ CFU/mL of colony counts with error bars representing standard deviation values for each time point′s replicate. The data was analyzed via two‐way ANOVA with post hoc Dunnett′s test for multiple comparisons. Asterisks denote statistically significant difference (*p* < 0.05) as compared with DMSO  ^∗^(*p*),  ^∗∗^(*p* = >0.01),  ^∗∗∗^(*p* > 0.001).

### 3.4. Evaluation of Maslinic Acid′s Cytotoxicity Against Colonic Epithelial Cells

Caco‐2 epithelial cells were challenged by maslinic acid at increasing concentrations to determine its cytotoxicity. Maslinic acid was tolerable to the cells up to the highest concentration tested (128 *μ*g/mL), with ~95% cell viability, as compared with the negative control group of cells incubated with 2% DMSO (Figure 2).

**Figure 2:**
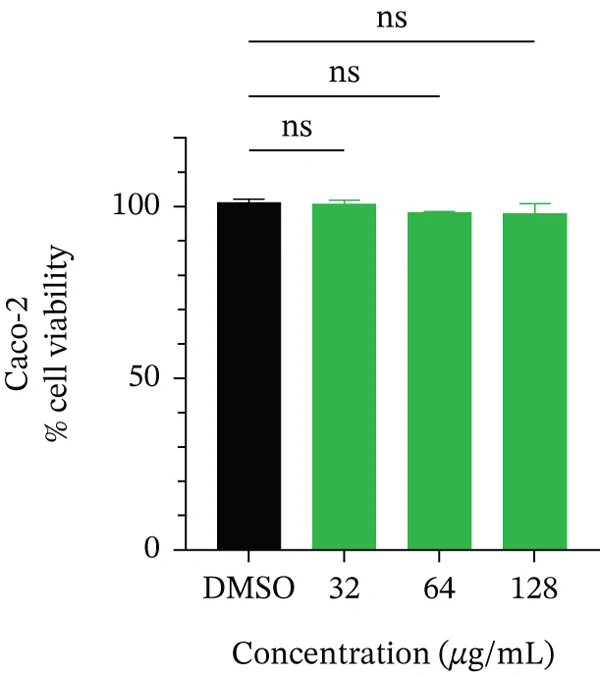
Cytotoxicity assessment of maslinic acid against Caco‐2 cells. Cells were incubated with maslinic acid for 24 h; 2% DMSO was used as a negative control. Results are shown as a percentage of cell viability relative to the DMSO control group. Error bars represent the standard deviation. The data were analyzed via one‐way ANOVA with post hoc Dunnett′s test for multiple comparison; ns, nonsignificant.

### 3.5. Inoculum Effect on the Anti‐*C. difficile* Activity of Maslinic Acid

Testing the effect of high inoculums on the antibacterial activity of new agents is an important consideration, particularly for *C. difficile* infections where bacterial clearance is vital for preventing recurrence. Therefore, maslinic acid′s activity against high concentrations of *C. difficile* (10^6^ and 10^7^ CFU/mL) was evaluated. The MICs of maslinic acid against the high inoculum sizes of both strains demonstrated little to no difference in MICs (8–16 *μ*g/mL) when compared with the standard bacterial concentration (10^5^ CFU/mL) (Table 3). This indicates that maslinic acid′s activity was not affected high *C. difficile* inoculum size. The MICs of the control antibiotics, vancomycin and fidaxomicin, against the high inoculum sizes displayed little to no change (MIC ranges of 0.5–2 and 0.015–0.06 *μ*g/mL for vancomycin and fidaxomicin, respectively).

**Table 3:** Assessment of the *C. difficile* inoculum effect on maslinic acid′s activity.

Test agents	*C. difficile* ATCC 43255			*C. difficile* ATCC‐BAA 1870		
	10^5^ CFU/mL	10^6^ CFU/mL	10^7^ CFU/mL	10^5^ CFU/mL	10^6^ CFU/mL	10^7^ CFU/mL
Maslinic acid	8	8	16	16	16	16
Vancomycin	0.5	0.5	0.5	1	2	2
Fidaxomicin	0.015	0.03	0.06	0.03	0.06	0.06

### 3.6. Activity of Maslinic Acid in SGF and SIF

To evaluate whether the activity of maslinic acid is deleteriously affected by the gastric and intestinal environments, maslinic acid was exposed to SGF and SIF for 4, 24 and 48 h, and its activity was evaluated against *C. difficile.* As shown in Table 4, after incubation with SGF for up to 48 h, the MICs of maslinic acid remained the same or increased by onefold only against both strains. This result suggests that maslinic acid was not affected by prolonged incubation with gastric pH or gastric enzymes. Similarly, vancomycin and fidaxomicin MICs remained the same or saw a slight onefold difference in activity.

**Table 4:** Assessment of maslinic acid activity against *C. difficile* when exposed to simulated gastric fluid (SGF).

Test agents	MICs (*μ*g/mL) after incubation with SGF							
	*C. difficile* ATCC 43255				*C. difficile* ATCC‐BAA 1870			
	0 h	4 h	24 h	48 h	0 h	4 h	24 h	48 h
Maslinic acid	8	8	8	8	16	16	16	16
Vancomycin	0.5	0.5	0.5	0.5	1	1	1	1
Fidaxomicin	0.03	0.03	0.03	0.03	0.06	0.06	0.06	0.06

In addition, after incubation with SIF for up to 48 h, the activity of maslinic acid was not affected (MICs = 16 * μ*g/mL) as compared with its activity without exposure to SIF (Table 5).Vancomycin′s and fidaxomicin′s activity were also not affected by incubation with SIF (MICs = 0.5–2 *μ*g/mL for vancomycin and MICs = 0.03–0.06 *μ*g/mL for fidaxomicin).

**Table 5:** Assessment of maslinic acid activity against *C. difficile* when exposed to simulated intestinal fluid (SIF).

Test agents	MICs (*μ*g/mL) after incubation with SIF							
	*C. difficile* ATCC 43255				*C. difficile* ATCC‐BAA 1870			
	0 h	4 h	24 h	48 h	0 h	4 h	24 h	48 h
Maslinic acid	16	16	16	16	16	16	16	16
Vancomycin	0.5	0.5	0.5	1	1	2	2	2
Fidaxomicin	0.03	0.03	0.03	0.03	0.03	0.06	0.06	0.06

### 3.7. Mechanistic Insights of Maslinic Acid

Compounds that have rapid bactericidal activity typically cause disruption or permeabilization of the bacterial cell membrane. To determine whether maslinic acid′s activity has these same effects, we evaluated its impact on *C. difficile* cell membrane integrity through a multitude of cell permeability assays (ATP, DNA, and protein leakage assays [Figure S1]). Nisin, a control antibiotic for increasing cell permeability [38], caused high concentrations of intracellular ATP to leak out into media (4 × 10^6^ relative luminescence units [RLU] in extracellular ATP), as well as DNA and protein (~100% leakage). Interestingly, in all assays, maslinic acid demonstrated no leakage of any of the three aforementioned components, with levels similar to that of the negative controls DMSO and antibiotic vancomycin. Despite its rapid killing activity seen in the time‐kill kinetics assay (Figure 1), maslinic acid did not induce cell membrane permeabilization and displayed no leakage of either intracellular ATP, DNA, or protein from *C. difficile* cells. Therefore, we sought to investigate the biosynthetic pathways of *C. difficile* that could be inhibited by maslinic acid via the macromolecular biosynthesis assays.

The incorporation of the radiolabeled precursors, ^14^C N‐acetylglucosamine into the cell wall, ^14^C leucine into protein, ^3^H thymidine into DNA, ^3^H uridine into RNA, and ^14^C acetic acid sodium into lipids was monitored. Oxacillin, linezolid, gatifloxacin, rifaximin, and triclosan were included as positive controls for peptidoglycan, protein, DNA, RNA, and lipid biosynthesis, respectively. Maslinic acid (at 1× and 2× MIC) significantly inhibited all the five biosynthetic pathways by approximately 65% to 95%. This effect is likely because of maslinic acid′s rapid bactericidal activity. Therefore, we sought to investigate the effect of maslinic acid at a lower concentration (0.5× MIC) on the biosynthesis pathways of *C. difficile.* Interestingly, maslinic acid (0.5× MIC) exhibited a pronounced inhibitory effect on protein and lipid biosynthesis, reducing the incorporation of the radiolabeled precursors by approximately 50%. This effect was followed by a slightly less pronounced inhibition of cell wall with incorporation of radiolabeled material at around 60%. DNA and RNA biosynthesis showed no inhibition with maslinic acid (0.5× MIC) as evidenced by the high percent of radioactive material incorporated (Figure 3). This data suggests that protein and lipid biosynthesis pathways could be the primary targets of maslinic acid. Yet, further mechanistic investigations are needed to meticulously define the intracellular bacterial target of maslinic acid which would be explored in our future studies.

**Figure 3:**
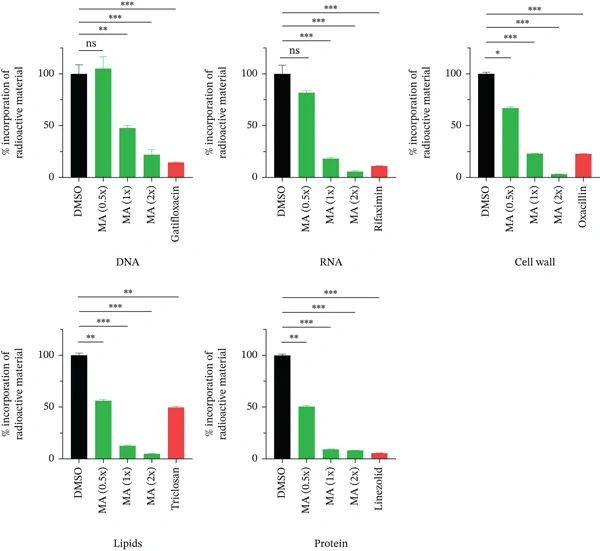
Macromolecular biosynthesis assays of maslinic acid‐treated *C. difficile. C. difficile*, at logarithmic phase, was treated with either maslinic acid (MA), 2% DMSO (negative control), or a control antibiotic targeting each biosynthesis pathway, and the incorporation of the radiolabeled precursors ^14^C N‐acetylglucosamine into the cell wall, ^14^C leucine into protein, ^3^H thymidine into DNA, ^3^H uridine into RNA, and ^14^C acetic acid sodium into lipids was quantified in *C. difficile* ATCC 43255. Oxacillin, linezolid, gatifloxacin, rifaximin, and triclosan were included as control antibiotics for cell wall, protein, DNA, RNA, and lipid biosynthesis pathways, respectively. The results are presented as percent incorporation of radioactive material for each pathway. The data were analyzed via one way ANOVA with post hoc Dunnett′s test for multiple comparisons. Asterisks denote statistically significant difference as compared with the negative control *p* < 0.05 ( ^∗^), *p* < 0.01 ( ^∗∗^), *p* < 0.001 ( ^∗∗∗^). Nonsignificant statistical differences are denoted by *p* > 0.05 (ns).

### 3.8. Activity of Structurally Similar Pentacyclic Triterpenes Against *C. difficile*

Finally, we investigated the antimicrobial activity of commercially available pentacyclic triterpene molecules (Table S3) that are structurally similar to maslinic acid (Table 6). The MICs of 32 pentacyclic triterpene compounds (Table S2) were determined against *C. difficile* ATCC‐BAA 1870. The compounds displayed moderate activity against *C. difficile*, with MICs spanning from 16 to > 128 *μ*M. The activity of maslinic acid was matched by several structurally related analogs including terminolic acid, bayogenin, gypsogenin, ilexgenin A, madecassic acid, rotundic acid, 18*β*‐glycyrrhetinic acid, asiatic acid, *β*‐boswellic acid, oleanolic acid, and polygalacic acid (MICs = 16 * μ*M). A moderate reduction in activity was observed for ursolic acid, corosolic acid, and 11‐keto‐*β*‐boswellic acid, with MICs of 32 *μ*M. Further decrease in activity was noted for compounds containing additional hydroxylations or stereochemical inversions such as 23‐hydroxybetulinic acid, pomolic acid, medicagenic acid, hederagenin, 3‐episursolic acid, and platicodigenin (MIC = 64 * μ*M). Ursonic acid, echinocystic acid, euscaphic acid, and 3‐epioleanolic acid—which lack the *β*‐oriented hydroxyl group at C‐3—were inactive against the strain tested (MIC = 128 * μ*M), further highlighting the importance of stereochemistry and functional group orientation. For example, oxidation of the C‐3 hydroxyl to a ketone (as in ursonic acid) or inversion of stereochemistry (3‐epimers) appears detrimental to activity, suggesting that the *β*‐oriented hydroxyl group at C‐3 is critical for maintaining antibacterial potency. Other molecules such as arjugenin, oleanonic acid, 18*α*‐glycyrrhetinic acid, *α*‐boswellic acid, epibetulinic acid, betulinic acid, polygalic acid, and quillaic acid demonstrated no anti‐*C. difficile* activity, with MIC values > 128 *μ*M (Table S2).

**Table 6:** MICs (*μ*M) of pentacyclic triterpene molecules structurally related to maslinic acid against *C. difficile* ATCC‐BAA 1870.

Compounds	MICs (*μ*g/mL)
Terminolic acid	16
Bayogenin	16
Gypsogenin	16
Ilexgenin A	16
Madecassic acid	16
Rotundic acid	16
18*β*‐Glycyrrhetinic acid	16
Asiatic acid	16
*β*‐Boswellic acid	16
Oleanolic acid	16
Polygalacic acid	16
Ursolic acid	32
Corosolic acid	32
11‐Keto‐*β*‐boswellic acid	32
23‐Hydroxybetulinic acid	64
Pomolic acid	64
Medicagenic acid	64
Hederagenin	64
3‐Episursolic acid	64
Platicodigenin	64
Ursonic acid	128
Echinocystic acid	128
Euscaphic acid	128
3‐Epioleanolic acid	128
Arjugenin	> 128
Oleanonic acid	> 128
18*α*‐Glycyrrhetinic acid	> 128
*α*‐Boswellic acid	> 128
Epibetulinic acid	> 128
Betulinic acid	> 128
Polygalic acid	> 128
Quillaic acid	> 128
Maslinic acid	16
Vancomycin	1
Fidaxomicin	0.03

## 4. Discussion

*C. difficile* infection remains a major healthcare challenge because treatment is frequently complicated by recurrence, disruption of the intestinal microbiota, and the limited number of effective therapeutic options. Consequently, there is continued interest in identifying agents that selectively target *C. difficile* while minimizing collateral effects on beneficial gut microorganisms. In this study, maslinic acid exhibited several characteristics that support its potential as a lead compound for *C. difficile* infection therapy, including selective antibacterial activity, rapid bactericidal action, low epithelial cytotoxicity, and stability under simulated gastrointestinal conditions.

Maslinic acid inhibited a diverse panel of *C. difficile* clinical isolates with MIC values ranging from 8 to 16 *μ*g/mL, including hypervirulent strains associated with increased disease severity and healthcare outbreaks [39]. Many successful antimicrobial agents originated from natural product scaffolds that were subsequently optimized through medicinal chemistry; the potency observed here provides a similar promising foundation for future structure activity relationship (SAR) studies [26, 40] .

An important characteristic of an ideal *C. difficile* therapeutic is preservation of the indigenous intestinal microbiota, which plays a central role in preventing *C. difficile* colonization and recurrence [41]. Broad‐spectrum antibiotics disrupt these microbial communities and diminish colonization resistance, thereby increasing susceptibility to recurrent infection [42]. In contrast to vancomycin and fidaxomicin, maslinic acid demonstrated relatively weak activity against representative species of *Lactobacillus*, *Bifidobacterium*, and *Bacteroides*. These organisms have been associated with restoration of intestinal homeostasis through mechanisms including nutrient competition, bile acid metabolism, and direct inhibition of *C. difficile* growth or toxin production [43]. *Bifidobacterium* in particular has been known to directly inhibit *C. difficile* and prevent infection through manipulation of bile acids critical for *C. difficile*′s germination and sporulation [44–46]. Additionally, *Bacteroides* strains were reported to contribute to bile acid‐mediated inhibition of *C. difficile* and prevent *C. difficile* infections [47]. Although only representative strains were evaluated, the observed selectivity suggests that maslinic acid may preserve beneficial microbial populations more effectively than currently available therapies. In contrast, vancomycin and fidaxomicin demonstrated potent activity against most intestinal microbiota strains tested, as reported previously [48, 49]. Time‐kill analysis further distinguished maslinic acid from the comparator antibiotics. Treatment reduced viable bacterial counts below the limit of detection within 2 h, whereas vancomycin and fidaxomicin required substantially longer to achieve complete killing, consistent with previous reports [31, 50]. Rapid elimination of vegetative cells is advantageous as it may shorten toxin production and reduce bacterial burden more quickly during infection, yet these possibilities remain to be evaluated in vivo.

One concern during antimicrobial development is whether activity declines under conditions that better reflect the infectious environment. For instance, elevated bacterial inoculum can reduce antibiotic efficacy by increasing the overall bacterial burden or altering physiological states within the population [51]. Maslinic acid maintained comparable MIC values across inoculum sizes ranging from 10^5^ to 10^7^ CFU/mL, indicating that increased cell density had little measurable impact on antibacterial activity. Similar findings were observed for fidaxomicin, in agreement with previous studies [52, 53].

Another favorable characteristic was the stability of maslinic acid under simulated gastrointestinal conditions. Because orally administered compounds intended to treat *C. difficile* infection must remain active throughout gastrointestinal transit, susceptibility to acidic pH or digestive enzymes represents a potential limitation [54]. Following exposure to SGF and SIF for up to 48 h, maslinic acid retained antibacterial activity with little or no change in MIC values. Vancomycin and fidaxomicin similarly remained stable under these conditions, consistent with published observations [52, 53]. Together with previous pharmacokinetic studies describing limited systemic absorption of maslinic acid [26, 27], these findings could support the potential feasibility of achieving therapeutically relevant luminal concentrations within the colon.

The mechanism responsible for the unusually rapid bactericidal activity of maslinic acid appears to differ from that of membrane‐active compounds. Agents that disrupt bacterial membranes typically induce leakage of intracellular ATP, nucleic acids, and proteins shortly after exposure [38]. Despite its rapid killing kinetics, maslinic acid produced no detectable release of these intracellular components (Figure S1A, S1B, S1C and S1D), indicating that bacterial membrane integrity remained largely intact. Instead, macromolecular biosynthesis assays demonstrated preferential inhibition of protein and lipid synthesis, whereas DNA and RNA synthesis were comparatively unaffected at subinhibitory concentrations. These findings suggest that intracellular biosynthetic pathways are more likely to underlie antibacterial activity than generalized membrane disruption. Nevertheless, future studies are warranted for identification of the direct molecular target using complementary approaches, including sequencing resistant mutants, target engagement studies, or chemical proteomics.

Minor alterations of chemical structures can affect the antibacterial activity of new therapies. Maslinic acid belongs to pentacyclic triterpenes family of compounds for which a wide array of compounds structurally related to maslinic acid are commercially available. SAR investigation could be key to developing maslinic acid analogs with greater potency against *C. difficile.* Additionally, maslinic acid has a small molecular weight (472.7 g/mol) which could make an interesting choice for future modifications to improve its efficacy in vitro and in vivo. Hence, we sought to evaluate the anti‐*C. difficile* activity of a library containing 32 pentacyclic triterpene compounds. Pentacyclic triterpenes structurally related to maslinic acid demonstrated a broad but structure‐dependent range of activity against *C. difficile*, with MICs spanning from 16 to > 128 *μ*g/mL. The activity of maslinic acid was matched by several structurally related analogs including terminolic acid, bayogenin, gypsogenin, ilexgenin A, madecassic acid, rotundic acid, 18*β*‐glycyrrhetinic acid, asiatic acid, *β*‐boswellic acid, oleanolic acid, and polygalacic acid (MICs = 16 * μ*g/mL). These compounds share a common pentacyclic scaffold (primarily oleanane or ursane types) with conserved features such as a C‐3 hydroxyl group and a C‐28 carboxylic acid, suggesting that this combination is favorable for antibacterial activity. The presence of additional hydroxyl substitutions, particularly at positions C‐2 or C‐23, as seen in maslinic acid and related compounds, does not significantly impair activity and may contribute to maintaining an optimal balance between polarity and membrane interaction. A moderate reduction in activity was observed for ursolic acid, corosolic acid, and 11‐keto‐*β*‐boswellic acid (MICs = 32 * μ*g/mL), indicating that subtle structural differences between oleanane and ursane frameworks, or the introduction of a keto group (e.g. at C‐11), can slightly diminish potency. Further decreases in activity were noted for compounds such as 23‐hydroxybetulinic acid, pomolic acid, medicagenic acid, hederagenin, 3‐episursolic acid, and platicodigenin (MICs = 64 *μ*g/mL). These derivatives often contain additional hydroxylations or stereochemical inversions (e.g. epimers at C‐3), which may disrupt optimal interactions with target sites, possibly by increasing polarity or altering three‐dimensional conformation. Compounds with MIC values of 128 *μ*g/mL, including ursonic, echinocystic, euscaphic, and 3‐epioleanolic acids, further highlight the importance of stereochemistry and functional group orientation. For example, oxidation of the C‐3 hydroxyl to a ketone (as in ursonic acid) or inversion of stereochemistry (3‐epimers) appears detrimental to activity, suggesting that the *β*‐oriented hydroxyl group at C‐3 is critical for maintaining antibacterial potency. Finally, compounds with MIC values > 128 *μ*g/mL, such as arjugenin, oleanonic acid, 18*α*‐glycyrrhetinic acid, *α*‐boswellic acid, epibetulinic acid, betulinic acid, polygalic acid, and quillaic acid, indicate that more substantial structural deviations, such as changes in ring framework (e.g. lupane vs. oleanane), stereochemical inversion at key positions (e.g. 18*α* vs 18*β*), or excessive oxidation, are unfavorable for activity against *C. difficile.*

It is worth mentioning that this study has three limitations. First, the therapeutic efficacy of maslinic acid needs to be evaluated in animal models of *C. difficile* infection. Second, only representative members of the intestinal microbiota were evaluated, and broader microbiome analyses will be necessary to determine whether maslinic acid truly preserves community diversity during treatment. Third, although the macromolecular biosynthesis assays narrowed the range of potential cellular targets, the precise molecular mechanism responsible for bacterial killing is yet to be deeply identified.

In conclusion, maslinic acid represents a promising natural product scaffold for the development of selective therapies against *C. difficile*. Its combination of activity against clinically relevant isolates, rapid bactericidal action, and limited activity against representative commensal bacteria, gastrointestinal stability, and favorable preliminary SAR studies distinguishes it from many naturally occurring antibacterial compounds. Future studies should focus on medicinal chemistry optimization, target identification, pharmacokinetic characterization, microbiome preservation in vivo, and efficacy testing in established *C. difficile* infection animal models.

## Author Contributions

Brice J. Stolz: Conceptualization (lead); writing—original draft (lead); formal analysis (lead); writing—review and editing (equal); investigation (lead); methodology (lead); validation (equal); and visualization (lead). Nader S. Abutaleb: Conceptualization (supporting); writing—original draft (supporting); formal analysis (supporting); writing—review and editing (equal); methodology (supporting); visualization (supporting); and supervision (supporting). Ahmed A. Abouelkhair: Conceptualization (supporting); writing—review and editing (supporting); and methodology (supporting). Mohamed N. Seleem: Writing—review and editing (supporting); supervision (lead); and resources (equal).

## Funding

No funding was received for this manuscript.

## Ethics Statement

The authors have nothing to report.

## Conflicts of Interest

The authors declare no conflicts of interest.

## Supporting information


**Supporting Information** Additional supporting information can be found online in the Supporting Information section. Table S1: Full description, sources, and characteristics of *C. difficile* strains. Table S2: Pentacyclic triterpene library category (cat) numbers and Chemical Abstract Service (CAS) registration numbers of each member. Table S3: Pentacyclic triterpene library category chemical structures. Figure S1: Negative results attempting to confirm rapid membrane loss and expulsion of internal cellular materials including (A,B) ATP, (C) DNA, and (D) proteins.

## Data Availability

The data that supports the findings of this study are available in the Supporting Information of this article.
