# Supplementary material for: Repurposing the Natural Product Maslinic Acid for Combating Clostridioides difficile Infections
Source: Int J Microbiol. 2026 Nov 2;2026:9364932. doi: 10.1155/ijm/9364932 (PMC13728334; doi:10.1155/ijm/9364932)
Supplement: Supplementary file 1 [file IJM-2026-9364932-s001.docx]

**Supplementary Table 1. Full description, sources, and characteristics of *C. difficile* strains.**

| No. | *C. difficile Strain ID* | Alternate Designation | Source | Characteristics |
| --- | --- | --- | --- | --- |
| 1 | **ATCC 43255** | *Clostridium difficile* Isolate VPI 10463 | Sourced from ATCC. Isolated from abdominal wound | Positive for *tcdA, tcdB*. Negative for *cdtB*. |
| 2 | **ATCC-BAA 1870** | *Clostridium difficile* Isolate 4118 | Sourced from ATCC. Clinical Isolate (Ribotype – 027) | Positive for *tcdA, tcdB,* and *cdtB* |
| 3 | **ATCC-BAA 1871** | *Clostridium difficile* strain 4111 | Sourced from ATCC. | Positive for *tcdA, tcdB*. Negative for *cdtB*. |
| 4 | **ATCC 9689** | *Clostridioides difficile* strain 90556-M6S | Sourced from ATCC. Reference strain. | Positive for *tcdA, tcdB*. Negative for *cdtB*. |
| 5 | **ATCC 70057** | *Clostridioidies difficile* strain VPI 11186 | Sourced from ATCC. | Negative for *tcdA, tcdB,* and *cdtB*. |
| 6 | **ATCC 630** | *Clostridium difficile* BAA-1382 | Sourced from ATCC. | Positive for *tcdA, tcdB*. Negative for *cdtB*. |
| 7 | **HM-88** | NAP07 (CDC#2007054) | Sourced from BEI. Reference genome for human microbiome project (HMP). | Negative for *tcdA, tcdB,* Negative for CDT. |
| 8 | **HM-89** | NAP08 (CDC#2007019) | Sourced from BEI. Reference genome for HMP. | Negative for *tcdA, tcdB,* Negative for CDT. |
| 9 | **HM-745** | 70-100-2010 | Sourced from BEI Resources. Isolated from a patient with diarrhea. Reference for HMP. | Positive for *tcdA, tcdB,* Negative for CDT. |
| 10 | **NR-32888** | *Clostridium difficile* P8 | Sourced from BEI Resources. Isolated from a human patient with CDI. | Positive for *tcdA, tcdB,* |
| 11 | **NR-32884** | *Clostridium difficile* P3 | Sourced from BEI Resources. Isolated from fecal material of a human patient with CDI. | Positive for *tcdA, tcdB,* |
| 12 | **NR-32897** | *Clostridium difficile* P21 | Soured from BEI Resources. Isolated from fecal material of a human patient with relapsing CDI. | Positive for *tcdA, tcdB,* |
| 13 | **NR 49288** | *Clostridium difficile* Isolate 20110870 | Sourced from BEI Resources.  Isolated from a young adult female with HA CDI. | Positive for *tcdA, tcdB, tcdC* and *cdtB*. |
| 14 | **NR 49308** | *Clostridium difficile* Isolate 20120166 | Sourced from BEI Resources.  Isolated from an elderly female with CA CDI. | Positive for *tcdA, tcdB, tcdC.* Negative for CDT. |
| 15 | **NR 49310** | *Clostridium difficile* Isolate 20110986 | Sourced from BEI Resources.  Isolated from a young male patient with CA CDI. | Positive for *tcdA, tcdB, tcdC.* Positive for CDT. |
| 16 | **AR 1078** | *Clostridium difficile* Isolate 1078 | Sourced from the CDC. Isolated from unknown patient in 2016, USA. | Positive for *tcdA, tcdB,* Negative for CDT. |
| 17 | **AR 1081** | *Clostridium difficile* Isolate 1076 | Sourced from the CDC. Isolated from unknown patient in 2016, USA. | Positive for *tcdA, tcdB,* Negative for CDT. |
| 18 | **AR 1082** | *Clostridium difficile* Isolate 1081 | Sourced from the CDC. Isolated from unknown patient in 2016, USA. | Positive for *tcdA, tcdB,* Negative for CDT. |
| 19 | **AR 1087** | *Clostridium difficile* Isolate 1087 | Sourced from the CDC. Isolated from unknown patient in 2016, USA. | Positive for *tcdA, tcdB,* Negative for CDT. |
| 20 | **AR 1093** | *Clostridium difficile* Isolate 1093 | Sourced from the CDC. Isolated from unknown patient in 2016, USA. | Positive for *tcdA, tcdB,* Positive for CDT. |
| 21 | **AR 1094** | *Clostridium difficile* Isolate 1094 | Sourced from the CDC. Isolated from unknown patient in 2016, USA. | Positive for *tcdA, tcdB,* Positive for CDT. |

**ATCC**: The American Type Culture Collection

**BEI Resources:** The Biodefense and Emerging Infections Resources Repository

**CDC:** The Center for Disease Control and Prevention

**HA:** Hospital acquired

**CA:** Community acquired

**HMP:** Human Microbiome Project

**Supplementary Table 2. Pentacyclic triterpene library category (cat) numbers and Chemical Abstract Service (CAS) registration numbers of each member.**

| **Compound** | **MCE CAT No.** | **CAS No.** |
| --- | --- | --- |
| Terminolic acid | HY-N7652 | 564-13-6 |
| Bayogenin | HY-N1932 | 6989-24-8 |
| Gypsogenin | HY-121382 | 465-99-6 |
| Ilexgenin A | HY-N6255 | 108524-94-3 |
| Madecassic acid | HY-N0569 | 4373-41-5 |
| Rotundic acid | HY-N2217 | 20137-37-5 |
| 18β-Glycyrrhetinic acid | HY-N0180 | 471-53-4 |
| Asiatic acid | HY-N0194 | 464-92-6 |
| β-Boswellic acid | HY-N2513 | 471-53-4 |
| Oleanolic acid | HY-N0156 | 508-02-1 |
| Polygalacic acid | HY-N0801 | 22338-71-2 |
| Ursolic acid | HY-N0140 | 77-52-1 |
| Corosolic acid | HY-N0280 | 4547-24-4 |
| 11-Keto-β-boswellic acid | HY-N2056 | 17019-92-0 |
| 23-Hydroxybetulinic acid | HY-N0566 | 85999-40-2 |
| Pomolic acid | HY-N6601 | 13849-91-7 |
| Medicagenic acid | HY-N2472 | 599-07-5 |
| Hederagenin | HY-N0256 | 465-99-6 |
| 3-Epiursolic Acid | HY-N4289 | 989-30-0 |
| Platicodigenin | HY-N1991 | 22327-82-8 |
| Ursonic acid | HY-N1486 | 6246-43-4 |
| Echinocystic acid | HY-N0271 | 510-30-5 |
| Euscaphic acid | HY-N2566 | 53155-25-2 |
| 3-Epioleanolic acid | HY-N4290 | 25499-90-5 |
| Arjugenin | HY-N4294 | 58880-25-4 |
| Oleanonic acid | HY-N1487 | 17990-42-0 |
| 18α-Glycyrrhetinic acid | HY-N0375 | 1449-05-4 |
| α-Boswellic acid | HY-N0611 | 471-66-9 |
| Epibetulinic acid | HY-N0223 | 38739-77-5 |
| Betulinic acid | HY-10529 | 472-15-1 |
| Polygalic acid | HY-N0801 | 1260-04-4 |
| Quillaic acid | HY-N0839 | 631-01-6 |
| **Maslinic Acid** | HY-N0629 | 4373-41-5 |

**Supplementary Table 3. Pentacyclic triterpene library category chemical structures**

| **Compound** | **Structure** |
| --- | --- |
| Terminolic acid |  |
| Bayogenin |  |
| Gypsogenin |  |
| Ilexgenin A |  |
| Madecassic acid |  |
| Rotundic acid |  |
| 18β-Glycyrrhetinic acid |  |
| Asiatic acid |  |
| β-Boswellic acid |  |
| Oleanolic acid |  |
| Polygalacic acid |  |
| Ursolic acid |  |
| Corosolic acid |  |
| 11-Keto-β-boswellic acid |  |
| 23-Hydroxybetulinic acid |  |
| Pomolic acid |  |
| Medicagenic acid |  |
| Hederagenin |  |
| 3-Epiursolic Acid |  |
| Platicodigenin |  |
| Ursonic acid |  |
| Echinocystic acid |  |
| Euscaphic acid |  |
| 3-Epioleanolic acid |  |
| Arjugenin |  |
| Oleanonic acid |  |
| 18α-Glycyrrhetinic acid |  |
| α-Boswellic acid |  |
| Epibetulinic acid |  |
| Betulinic acid |  |
| Polygalic acid |  |
| Quillaic acid |  |
| **Maslinic Acid** |  |

**ATP, DNA, and protein leakage assays investigating membrane disruption by maslinic acid**

To investigate maslinic acid’s potential mechanism of action acting on the bacterial membrane, a variety of membrane assays were utilized. First, an ATP leakage as was used as previously reported [1, 2]. To further confirm leakage a DNA and protein leakage assay was also performed. All assays begin with a high-inoculum of log-phase *C. difficile* ATCC 43255. These are then aliquoted out into microcentrifuge tubes and treated with maslinic acid, nisin, DMSO, or vancomycin (at 3× and 5× MIC). Treated solutions are then incubated for 2 hours at 37°C anaerobically.

For ATP leakage detection, incubated cultures were centrifuged at 10,000 ×g for 10 minutes. Supernatants were collected and pellets were resuspended in pre-reduced BHIS. The amounts of ATP in the supernatants (extracellular) and resuspended pellets (intracellular) were measured via BacTiter-Glo Microbial Cell Viability kit (Promega, Madison, Winsconsin), following the manufacturer’s instructions. Luminescence was detected via a Tecan Spark multimode microplate reader.

For the DNA leakage measurement, cultures were centrifuged at 6000 RPM for 10 minutes. Supernatants were collected and the amount of DNA (ng/µL) released extracellularly was measured using NanoDrop One (Thermo Scientific).

Protein leakage was evaluated via Pierce BCA protein assay kit (Thermo Scientific), following the manufacturer's instructions [3, 4]. The OD_562_ was measured using Tecan Spark microplate reader. The concentration of protein released extracellularly was calculated from the absorbance readings and the standard curve.

**Supplementary Figure 1. Effects of MA (5× MIC) on the permeability of the bacterial cell membrane of *C. difficile* ATCC 43255.** (**A** and **B**) ATP leakage assay of MA. *C. difficile* was exposed to MA, vancomycin or nisin (5× MIC), and the percentage of (**A**) extracellular or (**B**) intracellular ATP was calculated and plotted. The data are presented as average relative luminescence units (RLU) after each treatment. (**C**) DNA leakage and (**D**) protein leakage assays were also calculated to further evaluate leakage of internal cellular components. Bacterial cells were subjected to MA and the average amount of extracellular DNA and protein (ng/mL) was measured and normalized relative to the control drug nisin (100% DNA and protein leakage) was calculated. DMSO was included as a negative control. The data from the ATP, DNA and protein leakage assays were analyzed via one-way ANOVA followed by Dunnett’s test for multiple comparisons. Asterisks indicate a statistically significant difference between treatment with MA or nisin and treatment with DMSO (negative control). Asterisks denote statistical significant difference as compared to the negative control P < 0.05 (*), P < 0.01 (**), P < 0.001 (***). Non-significant statistical differences are denoted by P > 0.05 (ns).

**References**

1.	Wu X, Cherian PT, Lee RE, Hurdle JG. The membrane as a target for controlling hypervirulent Clostridium difficile infections. J Antimicrob Chemother. 2013;68(4):806-15. Epub 20121221. doi: 10.1093/jac/dks493. PubMed PMID: 23264511; PubMed Central PMCID: PMCPMC3695663.

2.	Abouelkhair AA, Abutaleb NS, Seleem MN. The antiarrhythmic drugs dronedarone and amiodarone exhibit potent in vitro and in vivo activity against Clostridioides difficile. Int J Antimicrob Agents. 2025;67(1):107649. Epub 20251027. doi: 10.1016/j.ijantimicag.2025.107649. PubMed PMID: 41161584; PubMed Central PMCID: PMCPMC12699489.

3.	Harnvoravongchai P, Chankhamhaengdecha S, Ounjai P, Singhakaew S, Boonthaworn K, Janvilisri T. Antimicrobial Effect of Asiatic Acid Against Clostridium difficile Is Associated With Disruption of Membrane Permeability. Front Microbiol. 2018;9:2125. Epub 20180907. doi: 10.3389/fmicb.2018.02125. PubMed PMID: 30245677; PubMed Central PMCID: PMCPMC6137100.

4.	Abouelkhair Ahmed A, Abutaleb Nader S, Stolz Brice J, Seleem Mohamed N. Repurposing azole antifungals as rapid bactericidal, membrane-disruptive therapeutics against Clostridioides difficile. Microbiology Spectrum. 2026;0(0):e02900-25. doi: 10.1128/spectrum.02900-25.
